# Diatom community structure in relation to environmental factors in human influenced rivers and streams in tropical Africa

**DOI:** 10.1371/journal.pone.0246043

**Published:** 2021-02-03

**Authors:** Tebkew Shibabaw, Abebe Beyene, Aymere Awoke, Mulat Tirfie, Muluken Azage, Ludwig Triest

**Affiliations:** 1 Department of Environmental Health, School of Public Health, Bahir Dar University, Bahir Dar, Ethiopia; 2 Department of Biology, Vrije Universiteit Brussel, Brussels, Belgium; 3 Department of Environmental Health Sciences and Technology, Jimma University, Jimma, Ethiopia; 4 Biological and Environmental Science and Engineering Division (BESE), Red Sea Research Center, King Abdullah University of Science and Technology (KAUST), Thuwal, Saudi Arabia; 5 Department of Nutrition and Dietetics, School of Public Health, Bahir Dar University, Bahir Dar, Ethiopia; INRA, FRANCE

## Abstract

The contemporaneous effect of natural and anthropogenic factors involved in a watershed contribution to the seasonal and spatial variation of diatom community composition is widely discussed in the scientific literature. Yet, there is a paucity of scientific evidence indicating the effect of these factors on diatoms in tropical African regions characterized by distinct dry and wet seasons and season associated human activities like rainfed agriculture are commonly practiced. We applied multivariate techniques to determine the spatio-temporal drivers of diatom assemblage and diatom species richness in human influenced rivers and streams in Ethiopia. We simultaneously collected water and diatom samples from 24 sampling points during the wet (July) and dry (February) seasons. Both water and diatom samples were processed following standard procedures. We identified 169 species belonging to 45 genera in the studied lotic systems. We found that both season and land use factors were important in defining diatom composition (PERMANOVA, p<0.05) and species richness (ANOVA, p<0.05) patterns. Diatom community composition was driven by conductivity, dissolved oxygen, pH, and turbidity parameters (Monte Carlo permutation test, p<0.05). Besides, diatom species richness was driven by dissolved oxygen, soluble reactive phosphorus, and turbidity (GLMM, p<0.05). The study highlighted physicochemical parameters influenced by seasonal variation and human activity determined the composition of diatoms. This implies that the unique feature of heavy rain during the rainy season in the region followed by extensive flooding aggravated by the steep slope from the highlands to the lowlands plays a major role in shaping the diatom autecology in the region. Therefore, in applying biomonitoring in such regions considering the effect of runoff and dilution is imperative.

## Introduction

Although water is the abundant resource on earth its natural quality is deteriorating from time to time mainly due to human pressure in different forms [1]. Agricultural intensification, urbanization, and industrialization as a result of human population growth and technological advancement, are the main factors responsible for water quality deterioration in many parts of the world [2,3] including Ethiopia [4,5].

Water pollution mainly due to human activities leads to many undesirable consequences on ecosystem as well as human health [6]. Agricultural runoff and wastes from urban areas that get their way into different aquatic systems could lead to changes in the physical and chemical characteristics of the receiving water bodies [7]. For instance, it can cause alteration of physicochemical parameters of water quality which in turn affects the assemblage of biological communities residing in the aquatic ecosystem [8]. Water pollution influences the abundance and diversity of macro-invertebrates, zooplanktons, phytoplankton and other aquatic organisms [9]. Diatoms (Bacillariophyta) are single cell, siliceous cell wall algae and are the principal component from the phytoplankton division [10]. They can exist in all aquatic ecosystems (marine, brackish, fresh waters) including in some moist terrestrial ecosystems [10,11]. Among the different algae groups, diatoms are the most abundant making them the most preferred food for water organisms. Diatoms alone contribute 20% of the global primary productivity hence playing a tremendous role in aquatic food web systems. These unicellular plankton organisms also facilitate biogeochemical processes for different elements particularly for global cycling of silica and carbon [10].

Literatures have shown that diatom communities are responsive to environmental variations in different aquatic ecosystems [10,12]. Diatoms have responded to variations in water chemistry, season, and physiography variables [13]. Studies conducted in different geographic regions of the world [14–17] including the limited studies of tropical and subtropical Africa [18–20] indicated that the community assemblages of diatoms were impacted by human induced water quality alterations. Anthropogenic pressure is among the high-level factors that drive diatom community composition in lotic systems [4,21–23]. In addition, diatoms are affected by temporal variation [24,25] since it governs the underlying controlling factors such as hydrological characteristics, biogeochemical processes, and precipitation-induced surface runoff in these systems [26]. This indicates diatom community dynamics is a function of natural (e.g. temporal variation) as well as anthropogenic (e.g. pollutant releasing) factors. A number of findings indicated that the contemporaneous effect of natural and anthropogenic factors involved in a watershed contributed to the seasonal and spatial variation of diatom community compositions [21,23–25,27]. This is because the concentration and transport pathway of many human-induced water pollutants are season-dependent [28]. For instance, the concentration of pollutants mainly from point sources could be reduced by the dilution effect of high precipitation during the high flow season [29]. Contrarily, precipitation-induced surface runoff could introduce different pollutants to the nearly available water sources [26]. Pollutants amount in the receiving water sources could even rise in areas where poor agricultural practices and improper urban waste management activities are prevalent [30,31]. These high-level factors (season and human influence) in turn affect proximate factors, e.g. physicochemical parameters, that directly influence diatom community structure [24]. As indicated by others, physicochemical variables influenced by seasonal variation and human pressure are the primary factors affecting diatoms [21,24,27,32,33]. Thus, assessing the impact of human activities on diatoms in tropical countries with distinct dry and wet seasons is essential for managing water resources effectively. Despite this fact, studies to disentangle such association between season-dependent environmental stressors and diatom community structure are very limited in tropical Africa including Ethiopia.

The Ethiopian economy is highly dependent on rain-fed agriculture in which the rainy season (from June to September) is the peak time for agricultural activities [34]. This phenomenon is not different for farmers living in the Gilgel Gibe watershed in Southwest Ethiopia [5]. Agricultural malpractices combined with hilly topography of the area, significantly contribute to water quality changes in this watershed. Pollution of surface water sources by agricultural runoff loaded with agrochemicals and sediment worsens during the rainy season in particular [30]. Additionally, untreated urban wastes (mainly from Jimma town) that contain high organic matter and nutrients are being continuously discharged into these aquatic systems [31]. Because of these factors, the physicochemical water quality and assemblage of different aquatic organisms have been altered in the watershed [5,30,31,35].

A number of studies conducted in tropical Africa indicated that human-induced water quality changes affected the assemblage of diatoms in different lotic systems [4,18,21,22,32,36–38]. However, studies showing the effect of human pressure on benthic diatom community composition at different seasons are scarce in this region. This is important because the influence of human activities on diatom community composition could be modified by seasonal variations as it governs different environmental and climate factors [26]. Therefore, this study aimed; 1) to determine the effect of human-induced land use variations on diatom composition during the dry and wet seasons and 2) to identify the main environmental factors that drive diatom community structure and species richness in human influenced rivers and streams in Southwest Ethiopia.

## Materials and methods

### Ethics statement

The field sampling was done in accordance with Ethiopian national and regional regulations and permission was not necessary to collect the data. The field studies involved neither endangered nor protected species.

### Study area

**Fig 1:**
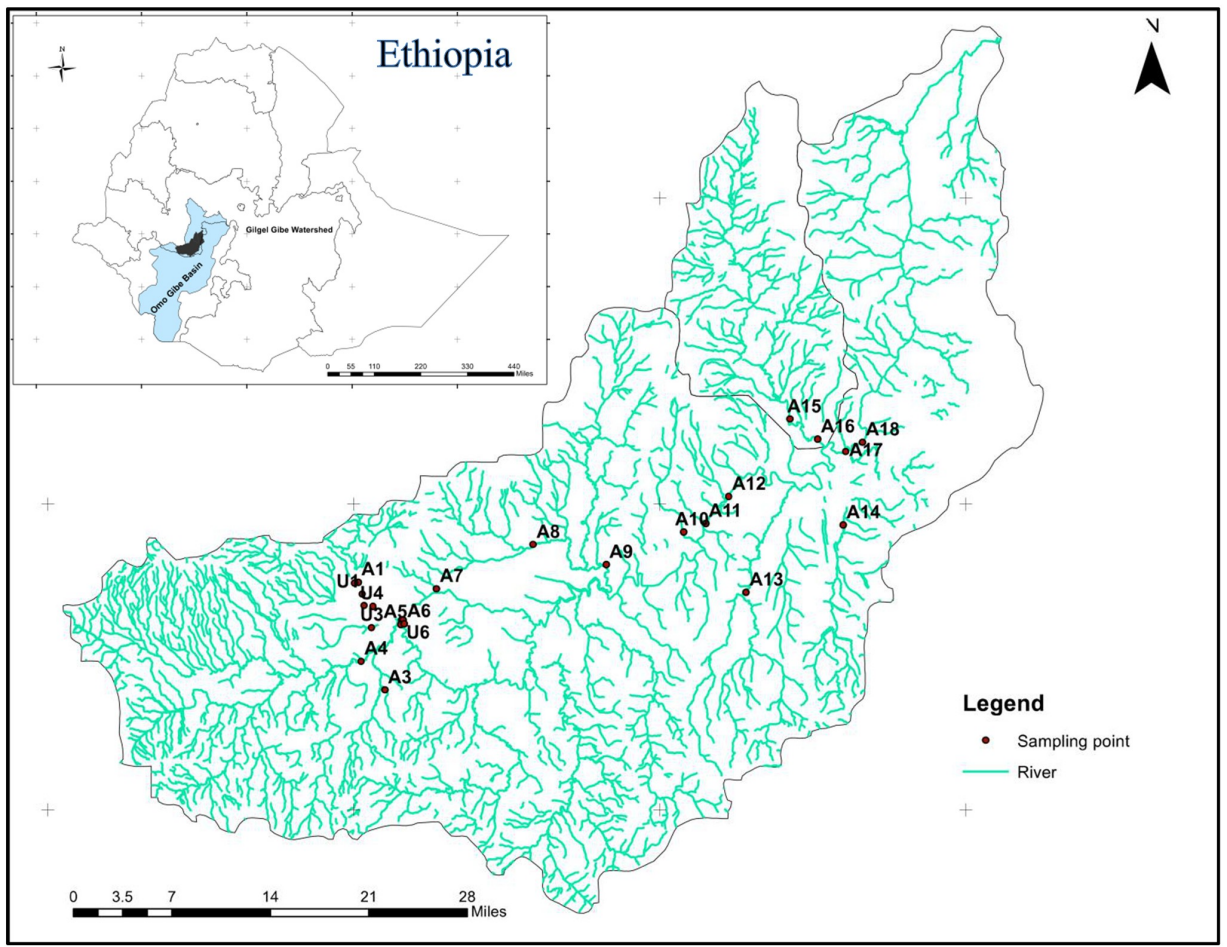
Sampling sites and river network in Gilgel Gibe watershed, Southwest Ethiopia. Reprinted from https://datacatalog.worldbank.org/dataset/ethiopia-rivers under a CC BY license, with permission from Ethiopia Central Statistics Agency (CSA), original copyright, 2013.

The study was conducted on rivers and streams draining in the Gilgel Gibe watershed in Southwest Ethiopia (Fig 1). In terms of climate, the watershed is characterized by a mean annual temperature ranged from 15°C to 22°C and a mean annual precipitation ranged from 1800 mm to 2300 mm. The minimum rainfall record is observed between December and February months, whereas the maximum rainfall is recorded between June and September [39]. Rivers and streams flowing in this watershed are the main contributors to feed the cascading Gilgel Gibe hydroelectric power dams constructed in the Omo-Gibe river basin. Water systems in Gilgel Gibe watershed are highly impacted by urban wastes with high organic load mainly from Jimma town and agricultural runoff containing soil particles and agrochemicals [30,31]. We collected water and diatom samples simultaneously from 24 sampling sites selected on five rivers and tributary streams. The samples were taken along Awetu (eight sites), Gilgel Gibe (ten sites), Nadaguda (two sites), Nedi (two sites), and Yedi (two sites) Rivers. Presence of human pressure in the form of farming (agricultural) and waste dumping (urban) was the criteria to select the sampling sites on the rivers and streams (tributaries). Hence, we recorded the general conditions (human influence, upper and adjust land uses) of the sampling sites using river assessment form. Based on the dominant land use/land cover type of the river reach (> 75%) before a sampling point, we grouped the sampling sites into urban impacted (sites within the influence of Jimma town) and agriculture impacted categories (see S1 Table for the sampling point’s characteristics). And to be either urban or agriculture impacted, there should be human activity (e.g. farming, waste dumping, settlement) within a reasonable distance (we considered 500 meters) adapted from a previous study conducted in the same watershed [40]. In this watershed system, the ratio of agriculture impacted sites is by far higher than the urban impacted sites. While there are many rivers and streams flowing in the watershed, it is only one river that is affected by urbanization. The other sites are dominantly affected by agricultural activity. Hence, we selected more agriculture impacted sampling sites [18] than urban impacted sites [6]. The samples were collected in July for the wet season and in February for the dry season.

### Water sampling and analysis

Water samples were collected for physicochemical parameters analysis at each sampling station. Representative samples were taken for each site by grabbing samples at three different random points within the site reach and were pooled together. Onsite measurements were done for dissolved oxygen (DO), water temperature, pH, and electrical conductivity using a multi-probe meter (Hach- Model-HQ30d Single-Input, Multi-Parameter Digital Meter). Turbidity was also measured using a Wag-WT3020 turbidity meter onsite. We filtered the water samples onsite using Whatman glass microfibre filters (25 mm Ø) and collected in polyethylene bottles of 100 ml for the analysis of nitrate, ammonia plus ammonium, and soluble reactive phosphorus. For the analysis of total dissolved solids (TDS), total phosphorus (TP), and total nitrogen (TN), well-mixed unfiltered water samples were collected using polyethylene bottles. All the water samples were then transported in a cold box to the laboratory of Environmental Health Sciences and Technology, Jimma University, Ethiopia where the analysis was performed. The samples were immediately put and kept in freezer until the analysis was performed. Both soluble reactive (SRP) and total phosphorus (TP) were measured using ascorbic acid methods, and total nitrogen (TN) was measured by Kjeldahl Nesseler method following American Public Health Association (APHA) [28]. High (0.3 to 30mg/l) and low (0.01 to 0.5 mg/l) ranges of nitrate were measured using the cadmium reduction method of 8039 and 8192, respectively. We also used Nesseler method 8038 for the determination of ammonia plus ammonium-nitrogen following the HATCH water analysis hand book of the US EPA, which is available online for free [41]. For TDS, we used gravimetric method as recommended by APHA protocol for water and waste water quality analysis [42].

### Diatom sampling

Diatom samples were collected from the available natural substrates (mainly stone and vegetation) by scrubbing the upper surface of the substrate using clean toothbrush. Three to five substrates were randomly selected with in 100m reach of the site, considering the micro habitat types, and scrubbed off until the total scrubbed surface area reaches approximately 100 square centimeters [4]. The aliquot diatom sample was transferred to falcon tubes and was preserved with 4% formalin (about 10% of the volume of the aliquot) [43]. The collected samples were transported to the laboratory for processing and identification.

### Diatom sample analysis and identification

For clear visualization of diatom frustules during identification, we used concentrated acid oxidation method for the cleaning of diatom frustules [44]. The samples were first pre-cleaned using concentrated hydrochloric acid (37%) by cooking gently on hot plate for about one hour to remove calcium. After frequent washing, the samples were cooked again using concentrated sulfuric (98%) and nitric (68%) acids consecutively taking one hour for each. After the digestion process was finished, we took 30 μL of aliquot sample from well-mixed and homogenized sample and we carefully pipetted it on a cleaned cover slip approximately making a circle. Once the samples were dried on the slide at room temperature, we mounted the cover slip with the slide using zyrax mounting medium on a warmed hotplate and dried it overnight to make it ready for identification.

Diatom frustules were identified and counted with a Carl Zeiss light microscope using a 100-x objective and only valves with more than half the intact valves within the field view were identified and counted. A total of about 300 and above diatom valves were identified and counted from each sample and then counts were converted into relative abundance (%). The identification was conducted to the minimum taxonomic level possible using standard taxonomic keys [45–49] and previous studies conducted in Africa [36,50,51].

### Data analysis

The physicochemical data did not follow normal distribution after normality test (Shapiro–Wilk test). Hence, we used Mann-Whitney U test to compare the variation in physicochemical parameters between agriculture and urban impacted sites. In addition, Wilcoxon signed rank test for paired samples was used to compare the difference in physicochemical measurements between the dry and wet seasons. In addition, two-way ANOVA was conducted to determine the effect of season and land use factors on species richness diversity measure. The analyses were performed in STATISTICA version 8.0 software [52]. Permutational Analysis of Variance (PERMANOVA) based on Bray-Curtis distance dissimilarity was performed on square-rooted diatom abundance data to see the effects of land use and seasonal variations on diatom community composition. Monte Carlo tests were performed with 9999 number of permutations. PERMANOVA+ in PRIMER version 7 was employed for the analysis [53].

Multivariate analysis using an ordination technique was used to explore the relation of environmental data with species data. Ordination analysis uses many variables to explore the degree of similarities or differences between samples or species [54]. Firstly, we run Detrended Canonical Analysis (DCA) on the species data of both seasons to determine the length of gradient. Since we found a length of gradients of 2.84SD, we run Redundancy Analysis (RDA). We first run the RDA with all physicochemical variables included to exclude the parameters that have high inflation factors (VIF >20). Inflation factor indicates the presence of collinearity among environmental variables [55]. A total of 53 diatom species observed in at least one sampling site with a relative abundance of ≥1% in one season, were included in the redundancy analysis. RDA with automatic forward selection option for environmental variables using Monte Carlo test with 999 unrestricted permutations was run to test statistical significance of each environmental variable in explaining species data structure [54]. For the analysis, species abundance data were transformed using Hellinger method [56]. Logarithmic transformation was applied on the environmental data (except pH) to reduce skewed distributions. CANOCO version 4.5 software [54] was used for the analysis.

Generalized linear mixed models (GLMMs) with an information-theoretic approach [57] were used to predict the effect of physicochemical parameters on diatom species richness. Sampling site was included in the models as a random factor to handle spatial autocorrelation. Models were run with Poisson link function, as the response variable was count data. Predictors except pH were log-transformed to improve model performance and interpretability [58]. Prior to the analysis, presence of collinearity effect among all predictors was identified by looking the variance inflation factors (VIFs). The *usdm* package in R software was used for VIF analysis. After excluding the predictors with high VIF, the full model was run by including all non-collinear predictors. Then, all possible models with different combinations of predictors included in the global model were built using the *dredge* function in *MuMin* package [59]. Second-order Akaike Information Criterion (AICc) was used to select the best model. The ‘top model set’ containing all models assumed to have comparable support in the data was selected using a ΔAICc cut-off of ≤6 [58]. If the ‘top model set’ consisted of more than one top model, the Akaike’s weights (AICw) were used to derive averaged models across all candidate top models in which a given predictor is present using the *model*.*avg* function. Linear regressions were done in the R software version 3.6.1 [60] using *lme4* package.

## Results

### Physicochemical characteristics of the sampling sites

The values of physicochemical parameters measured in this study are summarized in Table 1. The study indicated that conductivity, pH, turbidity, and nutrients (ammonia plus ammonium-N, nitrate, SRP, and TN) varied among seasons (Wilcoxon signed rank test, *p* < 0.05). Higher levels of nitrate, SRP, TN, and turbidity contaminants were recorded during the wet (high flow) season. Conversely, conductivity and pH concentrations were low during this season. We also confirmed that conductivity, DO, pH, and TDS amounts were found to be significantly different among land use types (Mann-Whitney U test, p <0.05). Lower concentration of DO was recorded in the urban sites. In contrast, conductivity and TDS measurements were high in the urban impacted sites. On the other side, high pH values were related to agricultural sites. Additional data on the measurement of physicochemical parameters can be found in S1 Table.

**Table 1:** Mean (range) values of the measured physicochemical characteristics of rivers and streams in the Gilgel Gibe watershed.

Parameters	Dry season		Wet season	
	AIS	UIS	AIS	UIS
Dissolved oxygen (mg/l)^**a**^	7.19 (5.70–12.52)	2.72 (0.22–6.03)	6.72 (5.08–7.89)	3.29 (0.57–6.83)
Temperature (°C)	20.9 (14.6–26.4)	22.0 (18.8–26.5)	20.4 (17.9–27.6)	21.3 (20.5–22.5)
Conductivity (μS/cm)^*^^a^	152.1 (84.6–440.0)	227.8 (113.2–500.0)	99.2 (62.0–367.0)	132.7 (78.0–254.0)
pH^*^^a^	8.4 (8.0–9.5)	7.2 (6.5–7.6)	7.5 (6.4–8.0)	6.6 (5.3–7.3)
Turbidity (NTU)^*^	36.0 (6.4–123.0)	21.2 (8.0–37.2)	159.6 (64.9–318.0)	61.9 (35.0–82.0)
Total dissolved solids (mg/l)^**a**^	102.7 (12.0–776.0)	426.7 (20.0–1236.0)	124.9 (32.0–296.0)	138.8 (80.0–305.0)
Total phosphorus (mg/l)	0.12 (0.05–0.51)	0.27 (0.04–1.01)	0.50 (0.07–2.34)	0.10 (0.03–0.25)
Soluble reactive phosphorus (mg/l)^*^	0.04 (0.02–0.14)	0.06 (0.02–0.14)	0.03 (0.01–0.16)	0.02 (0.01–0.05)
Ammonia + ammonium-N (mg/l)^*^	0.64 (0.42–1.02)	1.02 (0.54–2.07)	0.40 (0.29–0.70)	0.78 (0.25–2.72)
Nitrate (mg/l)^*^	0.31 (0.02–1.27)	0.38 (0.04–0.80)	0.99 (0.02–1.87)	0.58 (0.13–1.30)
Total nitrogen (mg/l)^*^	1.71 (0.55–3.28)	4.39 (0.69–14.11)	3.98 (0.84–9.80)	3.92 (1.60–8.90)

AIS = Agriculture impacted sites [18]; UIS = Urban impacted sites [6]

(*) indicate significant seasonal difference

(^**a**^) indicate significant difference among land use impacts; significance was considered at p < 0.05.

### Diatom community composition

In the present study, 169 species belonging to 45 genera were identified from all sampling sites. *Gomphonema* (15 species), *Navicula* (25 species), *Nitzschia* (20 species), and *Pinnularia* (17 species) were the dominant genera with the highest taxa richness recorded in the sampling sites during both seasons. *Achnanthidium minutissimum* (6.43%), *Gomphonema parvulum* (17.58%), *Gomphonema pumilum* (7.11%), and *Nitzschia palea* (16.47%) were the most dominant diatom taxa observed during both seasons. *Achnanthidium minutissimum* and *Gomphonema pumilum* were more abundant in the agricultural sites. Besides, the urban impacted sites were dominated by *Gomphonema parvulum* and *Nitzschia palea* species especially during the dry season (Table 2). The relative abundance of all diatoms observed at each sampling site dung both seasons can be found in S2 Table.

**Table 2:** The mean relative abundance of dominant species (above 1% of total abundance), mean (range) species richness of diatoms observed across the 24 sites in the Gilgel Gibe watershed and their saprobity (Sapr) and trophic state (Trop) status.

Species	Sapr	Trop	Dry season		Wet season	
			AIS	UIS	AIS	UIS
*Achnanthidium minutissimum* (Kützing) Czarnecki	β-meso	diff	5.1	1.7	12.7	6.2
*Achnanthidium subhudsonis* (Hustedt) H. Kobayasi			5.7		2.1	0.2
*Adlafia bryophila* (Petersen) Moser Lange-Bertalot & Metzeltin	olig	meso	4.0		0.1	0.5
*Adlafia minuscula* (Grunow) Lange-Bertalot	β-meso	oli	1.1		2.6	0.7
*Fragilaria ulna* (Nitzsch.) Lange-Bertalot	α-meso/poly	diff	0.3	0.4	2.0	5.5
*Gomphonema gracile* Ehrenberg	olig	meso	2.9	4.5	2.0	1.8
*Gomphonema minutum* (Ag.)Agardh	β-meso	eut	6.8	6.7	2.5	
*Gomphonema parvulum* (Kützing) Kützing	α-meso/poly	eut	13.5	28.5	11.4	16.9
*Gomphonema pumilum* (Grunow) Reichardt & Lange-Bertalot	_	diff	10.0		16.5	2.0
*Halamphora montana* (Krasske) Levkov			0.9	0.9	1.6	2.8
*Luticola mutica* (Kützing) D.G. Mann			1.5		1.6	1.7
*Navicula cryptocephala* Kützing	α-meso	diff	2.2	3.8	1.9	1.3
*Navicula gregaria* Donkin	α-meso	eut	2.4	0.1	2.1	1.0
*Navicula rostellata* Kützing			3.2	1.9	0.3	0.6
*Navicula schroeteri* Meister	β-meso	eut	7.3	2.5	2.2	
*Nitzschia palea* (Kützing) W.Smith	poly	hyper	6.2	21.0	7.9	30.8
*Pinnularia obscura* Krasske	olig	_	2.0		6.6	0.9
*Surirella angusta* Kützing	β-meso	eut	2.2	0.7	0.5	
**Richness**	** **	** **	29.4 (23–37)	18.5 (14–25)	22.3 (14–32)	24.2 (13–34)

AIS = agriculture impacted sites [18], UIS = urban impacted sites [6], βmeso = β-mesosaprobous, αmepo = α-meso/polysaprobous, αmeso = α-mesosaprobousand, poly = polysaprobous, olgo = oligosaprobous, diff = in different, meso = mesotrophic, oli = oligotrophic, eut = eutrophic, hyper = hypereutrophic.

### Influence of seasonal and land use factors on diatoms

PERMANOVA based on Bray-Curtis dissimilarity showed that diatom structure differed among the land use groups (PERMANOVA F = 5.181; p < 0.001). There was also seasonal variation in diatom community composition in the studied rivers and streams (PERMANOVA F = 2.666; p < 0.001). However, the interaction between seasonal and land use variables in defining diatom variation was not statistically significant (PERMANOVA F = 1.195; p = 0.233). Besides, the influence of seasonal and land use variables on diatoms was reflected on species richness. Two-way ANOVA outputs indicated that species richness was differed among seasons (ANOVA F = 5.483; p = 0.024) as well as land use types (ANOVA F = 5.615; p = 0.022). We also confirmed the interaction between these factors was highly significant in explaining species richness pattern (ANOVA F = 14.376; p <0.001). Species richness was high in the agricultural sites and in samples that were sampled during the dry season compared to their counter parts. Species richness of all sampling sites is presented in S2 Table.

### Environmental factors associated to diatom community variation

**Fig 2:**
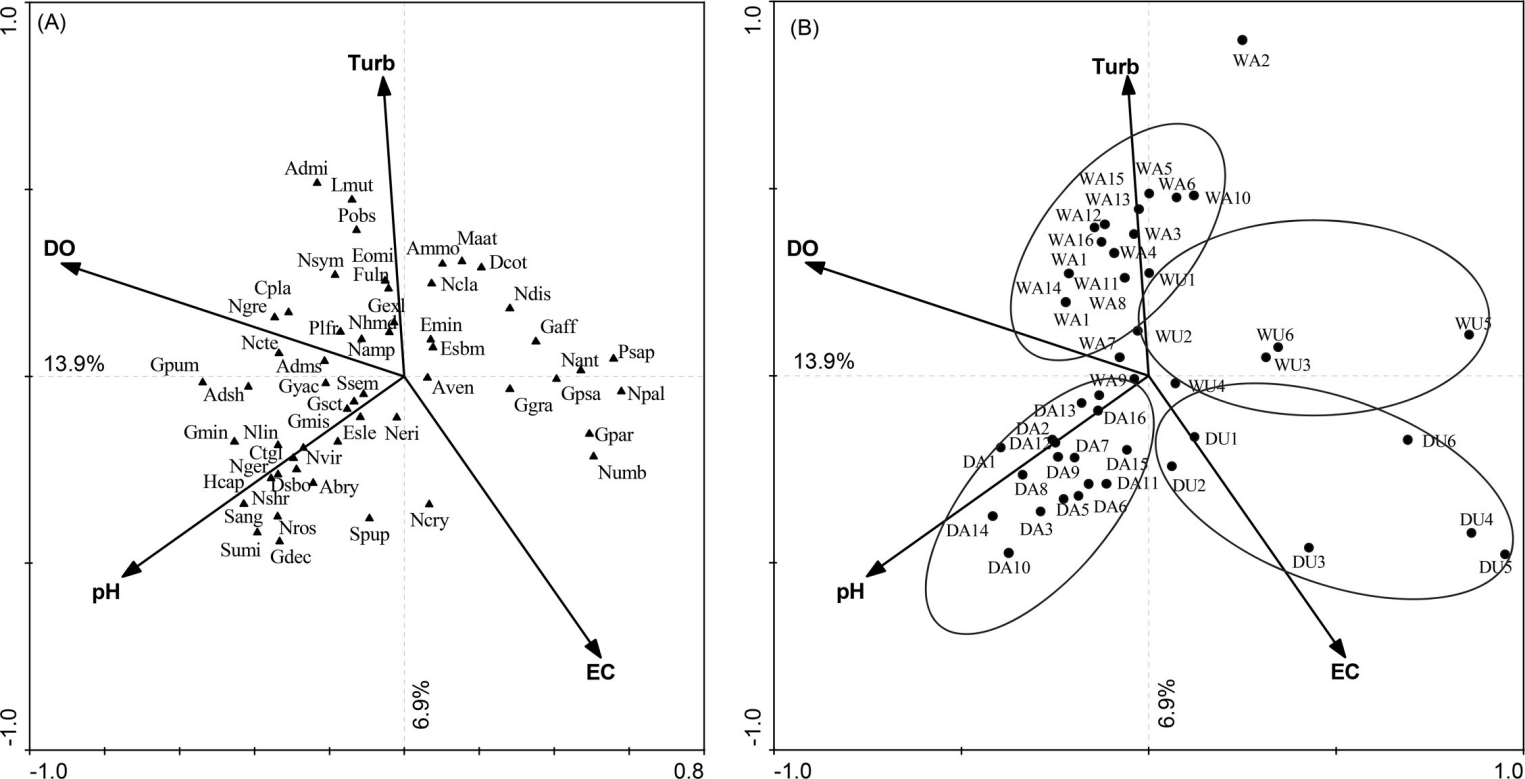
RDA ordination biplot diagram showing the relationship between measured significant environmental variables with (A) diatom species and (B) sample sites. Ellipses included in Fig 2B show site groupings. Abbreviations: DO–dissolved oxygen; EC–conductivity; Turb–turbidity; DA–dry agriculture; DU–dry urban; WA–wet agriculture; WU–wet urban. The full name of all diatom species is presented in Table 3.

Multivariate analysis using RDA ordination technique showed that 36.5% of the diatom community variation was captured by the measured environmental variables. Conductivity, dissolved oxygen, pH, and turbidity were the environmental variables that significantly explained diatom community distribution in the assessed lotic systems (Monte Carlo test, p < 0.050). The first two RDA axes explained 20.8% of diatom species variation (Fig 2). Axis 1, which is mainly constrained by dissolved oxygen, separated urban impacted sites from most agricultural sites. In addition, axis 2 that reflects a gradient of turbidity was positively associated with wet season sampled sites. This indicates both season and land use variables were important in defining diatom community distribution.

**Table 3:** List of diatom species codes presented in Fig 2 and their full names.

Code	Full name
ADMI	*Achnanthidium minutissimum* (Kützing) Czarnecki
ADSH	*Achnanthidium subhudsonis* (Hustedt) H. Kobayasi
ABRY	*Adlafia bryophila* (Petersen) Moser Lange-Bertalot & Metzeltin
ADMS	*Adlafia minuscula* (Grunow) Lange-Bertalot
AVEN	*Amphora veneta* Kützing
CPLA	*Cocconeis placentula* Ehrenberg
CTGL	*Cymbella turgidula* Grunow 1875 in A.Schmidt & al. var. turgidula
DCOT	*Diadesmis contenta* (Grunow ex V. Heurck) Mann
DSBO	*Diploneis subovalis* Cleve
ESLE	*Encyonema silesiacum* (Bleisch in Rabh.) D.G. Mann
EOMI	*Eolimna minima* (Grunow) Lange-Bertalot
ESBM	*Eolimna subminuscula* (Manguin) Moser Lange-Bertalot & Metzeltin
EMIN	*Eunotia minor* (Kützing) Grunow in Van Heurck
FULN	*Fragilaria ulna* (Nitzsch.) Lange-Bertalot
GDEC	*Geissleria decussis* (Ostrup) Lange-Bertalot & Metzeltin
GAFF	*Gomphonema affine* Kützing
GEXL	*Gomphonema exilissimum* (Grun.) Lange-Bertalot & Reichardt
GGRA	*Gomphonema gracile* Ehrenberg
GMIS	*Gomphonema minusculum* Krasske
GMIN	*Gomphonema minutum* (Ag.)Agardh
GPAR	*Gomphonema parvulum* (Kützing) Kützing
GPSA	*Gomphonema pseudoaugur* Lange-Bertalot
GPUM	*Gomphonema pumilum* (Grunow) Reichardt & Lange-Bertalot
GYAC	*Gyrosigma acuminatum* (Kützing)Rabenhorst
GSCA	*Gyrosigma scalproides* (Rabenhorst)Cleve
HLMO	*Halamphora montana* (Krasske) Levkov
HCAP	*Hippodonta capitata* (Ehr.)Lange-Bert.Metzeltin & Witkowski
LMUT	*Luticola mutica* (Kützing) D.G. Mann
MAAT	*Mayamaea atomus* (Kützing) Lange-Bertalot
NANT	*Navicula antonii* Lange-Bertalot
NCRY	*Navicula cryptocephala* Kützing
NCTE	*Navicula cryptotenella* Lange-Bertalot
NERI	*Navicula erifuga* Lange-Bertalot
NGER	*Navicula germainii* Wallace
NGRE	*Navicula gregaria* Donkin
NHMD	*Navicula heimansioides* Lange-Bertalot
NROS	*Navicula rostellata* Kützing
NSHR	*Navicula schroeteri* Meister
NSYM	*Navicula symmetrica* Patrick
NVIR	*Navicula viridula* (Kützing) Ehrenberg
NAMP	*Nitzschia amphibia* Grunow f.amphibia
NCLA	*Nitzschia clausii* Hantzsch
NDIS	*Nitzschia dissipata* (Kützing)Grunow
NLIN	*Nitzschia linearis* (Agardh) W.M.Smith
NPAL	*Nitzschia palea* (Kützing) W.Smith
NUMB	*Nitzschia umbonata* (Ehrenberg)Lange-Bertalot
POBS	*Pinnularia obscura* Krasske
PSAP	*Pinnularia saprophila* Lange-Bertalot. Kobayasi & Krammer
PLFR	*Planothidium frequentissimum* (Lange-Bertalot)Lange-Bertalot
SPUP	*Sellaphora pupula* (Kützing) Mereschkowksy
SSEM	*Sellaphora seminulum* (Grunow) D.G. Mann
SANG	*Surirella angusta* Kützing
SUMI	*Surirella minuta* Brebisson

Based on the RDA findings, three groups of sites were observed because of differences in species composition. The first group consisted of urban impacted sites of both seasons that are characterized by low dissolved oxygen and high conductivity. These sites were dominated by diatom species known to tolerate pollution such as *Gomphonema parvulum*, *Gomphonema pseudoaugur*, *Gomphonema gracile*, *Nitzschia palea*, and *Nitzschia umbonata*. The second group was composed of agricultural sites sampled during the dry season that were characterized by high pH and low turbidity amounts. *Achnanthidium subhudsonis*, *Adlafia bryophila*, *Diploneis subovalis*, *Geissleria decussis*, *Gomphonema minutum*, *Gomphonema pumilum*, *Navicula rostellata*, *Navicula schroeteri*, *Surirella angusta*, and *Surirella minuta* diatom species were abundant in these sites. The last group was composed of agricultural sites sampled during the wet season and depicted on upper part of the RDA plot. These sites were associated with high turbidity because of agricultural runoff loaded with soil particles and other sediments. These sites were dominated by *Achnanthidium minutissimum*, *Eolimna minima*, *Halamphora Montana*, *Luticola mutica*, *Navicula symmetrica*, and *Pinnularia obscura* diatom taxa (Fig 2).

GLMMs analysis produced 57 top candidate models (ΔAICc ≤6) with different variable combinations that were best in explaining species richness pattern. The regression results showed that DO, SRP, and turbidity variables were found to be significant predictors of diatom species richness in the studied waters (GLMM, p<0.05). The significant variables explained 45.8% (*Pseudo R*^*2*^*m* = 0.458) of the variation in species richness from the total 49.7% (*Pseudo R*^*2*^*m* = 0.497) that was captured by the measured physicochemical parameters. Species richness had a positive relation with dissolved oxygen. In contrast, turbidity and SRP had adverse effects on diatom diversity improvement (Table 4).

**Table 4:** Results of the generalized linear mixed models (GLMMs) analysis of diatom species richness as function of physicochemical predictors. Variable importance indicates the selection probability of each independent variable. Only significant variables (p<0.050) selected across all candidate models are presented.

Variable	Model-averaged coefficient	Adjusted SE	Variable importance
Intercept	3.115	0.569	
DO	0.553	0.144	1.00
Soluble reactive phosphorus	-0.181	0.123	0.50
Turbidity	-0.187	0.094	0.65

## Discussion

Studies indicating the influence of distal and proximate factors governed by natural and anthropogenic processes on diatom community structure and species richness are still scarce in tropical Africa compared with other regions. The present study assessed the influence of seasonal and land use variations, and environmental factors in dictating diatom composition and structure in rivers and streams in Southwest Ethiopia.

### Influence of temporal and land use factors on diatoms

A number of studies showed the influence of temporal and land use pattern factors on diatom community structure and composition in lotic systems [24,25,33,61–63] in support of our study. These high-level factors control proximate determinants (e.g. physicochemical parameters, flood, velocity, water level, etc) that directly influence these organisms [64]. As indicated by PERMANOVA, land use variation defined diatom composition in agreement with other studies [21,63]. The occurrence and abundance of some dominant species were related to a specific land use (Table 2). For instance, organic pollution and nutrient tolerant species such as *Gomphonema gracile*, *Gomphonema parvulum*, and *Nitzschia palea* [65] dominated in oxygen depleted urban sites, which receive untreated organic wastes generated by residents, institutions and industries of Jimma town as noted also by Haddis *et al*. [31]. On the other side, *Achnanthidium minutissimum*, *Achnanthidium subhudsonis*, *Gomphonema pumilum*, and *Navicula schroeteri* species were more dominant in the agriculturally impacted sites. Human impact variation in the form of agricultural activity and urbanization was an important factor in influencing diatom community composition [62,63,66]. The influence of land use is also supported by our RDA results in which urban impacted sites of both seasons were separated from agricultural sites based on dominant diatom species composition (Fig 2). Besides, land use impact difference was important in influencing species richness spatial pattern in the studied water systems. We found low richness in the urban impacted sites in agreement with other studies done in tropical, temperate, and boreal regions [19,33,63,67]. The reason for species richness variation among land uses could be that diatom species response to environmental variables is different as they have different optima and tolerance [68,69]. Hence, low species richness may indicate presence of hostile environment (chemical and physical pressure) unsuitable for occurrence of diatoms with narrow environmental optima. Consequently, only diatoms with broad range of tolerance to environmental conditions would exist in that specific site [70].

In this study, we also affirmed the influence of seasonal factor on diatoms as precipitation-induced surface runoff brought shifts in community composition similar to a study by Tan *et al*. [24]. Species composition changes observed more in the agricultural sites. Agricultural runoff facilitated by steep slope topography of the area transported high loads of sediment and agrochemical pollutants from the surroundings of sampled water systems [30]. This resulted increase in concentrations of turbidity and nutrients in the receiving rivers and streams, which might be responsible for temporal shift in diatom composition. For instance, species that dominated the agricultural sites during the wet season like *Pinnularia obscura*, *Luticola mutica*, and *Halamphora montana* were succeeded by *Achnanthidium subhudsonis*, *Navicula rostellata*, and *Navicula schroeteri* species during the dry season (Table 2). Studies conducted in Ethiopia and Kenya have recorded the latter group of species in less impacted clean waters enriched with oxygen [19,36,71]. Season was also found to be important determinant of diatom species richness pattern similar to a previous study done in China [24]. This community composition measure was significantly low during the wet season, again might be related to high nutrient and turbidity amounts that both could affect occurrence and growth of diatoms. Turbidity can affect community succession by obscuring sunlight, an important energy source for most algal community including diatoms [72]. Similarly, although nutrients are essential resources for algal growth [72], species richness peaks at intermediate nutrient concentrations and declines towards either of extreme concentrations [73]. Another possible explanation for the decrease in species richness during the high flow season could be erosion effect of seasonal flood, which affects diatom community succession. Seasonal flood can affect diatoms that have standing growth form leading to decrease in abundance and diversity [25].

### Diatom community structure in relation to environmental variables

Our study showed that physicochemical variables influenced by temporal and human impact factors were important in defining the community composition of diatoms similar to previous findings [24,25,33,38,61]. RDA results showed that the measured environmental variables explained 36.5% of the diatom community variation (Fig 2). The study confirmed conductivity, dissolved oxygen, pH, and turbidity were important in determining diatom community structure in the assessed rivers and streams (Monte Carlo test, p < 0.05).

We demonstrated the significant contribution of dissolved oxygen in structuring diatom communities, which is in agreement with other studies [4,37,63,71]. The influence of DO in our study could be related to presence of high organic matter in the urban sites. Decomposers consume the available dissolved oxygen in order to degrade the complex organic matter, which in turn might lead to anoxic condition in the urban sites. This created spatial variability of dissolved oxygen, which is an essential element for metabolism. Conductivity associated with urban waste discharging also affected diatom community composition as identified by other researchers [19,37,71]. Conductivity reflects ions that may influence nutrient availability and uptake by diatoms during primary production [74]. UV radiation affects diatom composition [72] by inhibiting diatom growth and production especially in translucent water bodies, which contain high-suspended particulate matter that absorbs UV radiation [75]. This indicates the effect of turbidity on diatom structure could be by influencing UV radiation that reach to substrates where diatoms develop on. A previous study also demonstrated the role of turbidity in diatom variation [33]. Moreover, in accordance with other studies [38,76], pH was found to be an important factor for diatoms. pH has a strong effect on diatom growth through either directly by causing physiological stress on diatoms [77], or indirectly by influencing other physicochemical variables.

Based on RDA grouping of sites, both season and land use variables were determinant factors for diatom community structure (Fig 2). Agricultural sites with high pH and DO were dominated by *Achnanthidium subhudsonis*, *Adlafia bryophila*, *Gomphonema minutum*, *Gomphonema pumilum*, *Navicula rostellata*, *Navicula schroeteri*, and *Surirella minuta* species during the wet season. These species also occurred in minimally impacted oligo to mesotrophic waters enriched with oxygen [36,71]. *Achnanthidium minutissimum*, *Pinnularia obscura*, *Halamphora montana*, and *Luticola mutica* species occurred in highly turbid waters [78]. Furthermore, species known for their tolerance to pollution such as *Gomphonema gracile*, *Gomphonema parvulum*, *Gomphonema pseudoaugur*, *Nitzschia palea*, and *Nitzschia umbonata* [65] abundantly occurred in the urban sites influenced by untreated waste dumping from Jimma town. These species frequently observed in oxygen poor waters with high conductivity previously [37,71,79].

Physicochemical variables are the main drivers of diatom species richness in lotic systems [33,67,80,81]. Our GLMMs findings showed DO, SRP, and turbidity variables were important in driving species richness in the assessed flowing waters (Table 3). Our results agreed with the study by Dalu *et al*. in which DO and turbidity were the principal predictors of diatom species richness in an Austal temperate rivers in South Africa [33]. Diatom species richness improves as DO level increases consistent with previous studies [33,80]. In contrast, turbidity was found to be a negative predictor of species richness. The effect of turbidity on diatoms may be indirect through reducing the photic zone, which is a limiting factor for chlorophyll *α* production. In addition, soluble reactive phosphorus affected diatom richness adversely. The negative effect of SRP on our diatoms could be related to nutrient enrichment, since it is the main precursor of eutrophication. Water systems highly enriched with phosphorus nutrient harbor less species richness [73].

## Conclusions

The study indicated that both seasonal and land use factors were important in defining diatoms, as diatom assemblage and species richness differed among the dry and wet seasons as well as the urban and agriculture impacted sites. The study also highlighted the significance of human induced land use variation in influencing physicochemical drivers of diatoms such as conductivity, dissolved oxygen, pH, and SRP. Moreover, the physicochemical parameters were observed to significantly change between season in a unique pattern that was attributed to the heavy rain during the wet season coupled with the steep slope of the landscape and a prolonged dry season. We found this condition very important in determining the diatom autecology to use diatoms as biomonitoring agents in water quality determination in the region. This would have an implication in developing tools to monitor water resources influenced by such unique season-dependent human activities.

## Supporting information

S1 Table Study site characteristics and physicochemical water quality measurements. (DOCX)

S2 Table Relative abundance of diatoms identified in all sampling sites during both seasons. (XLSX)
