# Supplementary material for: Diatom community structure in relation to environmental factors in human influenced rivers and streams in tropical Africa
Source: PLoS One. 2021 Feb 3;16(2):e0246043. doi: 10.1371/journal.pone.0246043 (PMC7857570; doi:10.1371/journal.pone.0246043)
Supplement: S1 Table — (DOCX) [file pone.0246043.s001.docx]

**S1 Table 1.** Study site characteristics

| **Site code** | **River/stream name** | **Altitude (masl)** | **Coordinates (UTM)** | | **Dominant land use (>75%)** |
| --- | --- | --- | --- | --- | --- |
|  |  |  | Northing | Easting |  |
| A1 | Awetu | 1727 | 260535 | 851024 | Agriculture |
| A2 | Shenkore | 1728 | 260101 | 850939 | Agriculture |
| U1 | Awetu | 1722 | 261013 | 849660 | Urban |
| U2 | Awetu | 1717 | 261208 | 848341 | Urban |
| U3 | Awetu | 1709 | 262033 | 845823 | Urban |
| U4 | Awetu | 1722 | 262206 | 848272 | Urban |
| U5 | Boye | 1706 | 265483 | 846681 | Urban |
| U6 | Boye | 1698 | 265645 | 846690 | Urban |
| A3 | Offole | 1753 | 263567 | 838674 | Agriculture |
| A4 | Gilgel Gibe | 1707 | 260853 | 841970 | Agriculture |
| A5 | Gilgel Gibe | 1703 | 265378 | 846179 | Agriculture |
| A6 | Gilgel Gibe | 1702 | 265763 | 846274 | Agriculture |
| A7 | Merewa | 1782 | 269483 | 850273 | Agriculture |
| A8 | Kersa | 1745 | 280530 | 855326 | Agriculture |
| A9 | Bulbul | 1712 | 288898 | 853049 | Agriculture |
| A10 | Arer | 1718 | 297758 | 856752 | Agriculture |
| A11 | Gilgel Gibe | 1706 | 300272 | 857738 | Agriculture |
| A13 | Nada Guda | 1701 | 304889 | 849855 | Agriculture |
| A14 | Yedhi | 1704 | 316002 | 857556 | Agriculture |
| A15 | Nedhi | 1718 | 309906 | 869680 | Agriculture |
| A17 | Gilgel Gibe | 1619 | 316304 | 865959 | Agriculture |
| A18 | Gilgel Gibe | 1590 | 318206 | 867038 | Agriculture |
| A12 | Gilgel Gibe | 1678 | 302907 | 860844 | Agriculture |
| A16 | Nedhi | 1671 | 313077 | 867405 | Agriculture |

**S1 Table 2.** Physicochemical water quality of the sampling sites during the dry season (n=24)

| Site | DO (mg/l) | WT (°C) | EC (µS/cm) | pH | Turbidity (NTU) | TDS (mg/L) | TP (mg/) | SRP (mg/l) | (NH₃+NH₄⁺)-N (mg/l) | Nitrate (mg/l) | TN (mg/l) |
| --- | --- | --- | --- | --- | --- | --- | --- | --- | --- | --- | --- |
| A1 | 7.44 | 18.9 | 84.6 | 8.3 | 22.8 | 168 | 0.09 | 0.02 | 0.6 | 0.22 | 1.38 |
| A2 | 6.51 | 19.7 | 104.2 | 7.98 | 21.4 | 28 | 0.06 | 0.02 | 0.42 | 0.47 | 1.62 |
| U1 | 6.03 | 22.6 | 113.2 | 7.49 | 21.1 | 20 | 0.07 | 0.03 | 0.54 | 0.26 | 1.65 |
| U2 | 3.6 | 21.2 | 155.5 | 7.57 | 35.6 | 712 | 0.27 | 0.1 | 0.78 | 0.8 | 4.12 |
| U3 | 1.16 | 18.8 | 206.3 | 7.47 | 16.1 | 96 | 0.16 | 0.14 | 1.45 | 0.52 | 4.65 |
| U4 | 1.05 | 26.5 | 500 | 6.93 | 37.2 | 308 | 1.01 | 0.06 | 2.07 | 0.6 | 14.11 |
| U5 | 0.22 | 20.5 | 201.8 | 6.5 | 8.01 | 1236 | 0.06 | 0.02 | 0.68 | 0.04 | 1.11 |
| U6 | 4.23 | 22.1 | 190.1 | 7.19 | 8.9 | 188 | 0.04 | 0.02 | 0.59 | 0.05 | 0.69 |
| A3 | 7.68 | 14.6 | 161.4 | 8.24 | 22.1 | 12 | 0.09 | 0.03 | 0.59 | 0.28 | 1.38 |
| A4 | 7.25 | 17.3 | 139.5 | 8.38 | 19.2 | 28 | 0.06 | 0.03 | 0.52 | 0.02 | 2.39 |
| A5 | 6.08 | 21.5 | 143.4 | 8.23 | 24 | 776 | 0.1 | 0.04 | 0.47 | 0.22 | 1.18 |
| A6 | 6.67 | 20.6 | 159 | 8.09 | 24.1 | 20 | 0.11 | 0.06 | 0.57 | 0.15 | 1.08 |
| A7 | 6.07 | 21.7 | 118.3 | 8.16 | 31.7 | 32 | 0.09 | 0.04 | 0.75 | 1.27 | 3.28 |
| A8 | 7.29 | 22.5 | 99.1 | 8.79 | 60.4 | 112 | 0.1 | 0.02 | 1.02 | 0.37 | 2.49 |
| A9 | 6.69 | 19.1 | 111.3 | 8.4 | 46.7 | 72 | 0.11 | 0.05 | 0.46 | 0.02 | 1 |
| A10 | 7.82 | 20.01 | 142.9 | 8.67 | 15.48 | 64 | 0.06 | 0.03 | 0.7 | 0.12 | 1.46 |
| A11 | 5.7 | 20.6 | 157.6 | 7.98 | 21.7 | 72 | 0.08 | 0.03 | 0.62 | 0.3 | 1.62 |
| A13 | 6.82 | 17.8 | 111.7 | 7.98 | 21.8 | 24 | 0.08 | 0.03 | 0.66 | 0.07 | 1.17 |
| A14 | 7.34 | 18.1 | 110.3 | 8.21 | 82.6 | 48 | 0.11 | 0.04 | 0.93 | 0.62 | 2.53 |
| A15 | 7.36 | 22.8 | 100.8 | 8.86 | 30 | 40 | 0.1 | 0.03 | 0.76 | 0.31 | 1.77 |
| A17 | 12.52 | 25.5 | 440 | 9.46 | 7.08 | 128 | 0.05 | 0.02 | 0.43 | 0.04 | 0.55 |
| A18 | 8.41 | 26.4 | 300 | 9.19 | 6.44 | 152 | 0.05 | 0.14 | 0.66 | 0.02 | 0.91 |
| A12 | 6.06 | 24 | 154.1 | 8.18 | 66.7 | 36 | 0.51 | 0.04 | 0.56 | 0.39 | 1.74 |
| A16 | 5.76 | 25.3 | 100.1 | 8.4 | 123 | 36 | 0.29 | 0.03 | 0.85 | 0.63 | 3.27 |

**S1 Table 3.** Physicochemical water quality of the sampling sites during the wet season (n=24)

| Site | DO (mg/l) | WT (°C) | EC (µS/cm) | pH | Turbidity (NTU) | TDS (mg/L) | TP (mg/l) | SRP (mg/l) | (NH₃+NH₄⁺)-N (mg/l) | Nitrate (mg/l) | TN (mg/l) |
| --- | --- | --- | --- | --- | --- | --- | --- | --- | --- | --- | --- |
| A1 | 7.58 | 19 | 63 | 7.62 | 100 | 96 | 0.07 | 0.02 | 0.33 | 0.99 | 2.96 |
| A2 | 6.13 | 20.1 | 93 | 7.04 | 64.9 | 84 | 0.07 | 0.01 | 0.36 | 0.91 | 3.55 |
| U1 | 6.83 | 21.8 | 78 | 5.28 | 77.6 | 132 | 0.14 | 0.02 | 0.25 | 1.15 | 3.79 |
| U2 | 6.75 | 20.9 | 86 | 7.26 | 77.9 | 96 | 0.03 | 0.01 | 0.46 | 1.3 | 4.34 |
| U3 | 2.04 | 22.5 | 115 | 6.85 | 35 | 100 | 0.05 | 0.01 | 0.39 | 0.38 | 2.39 |
| U4 | 1.27 | 20.5 | 254 | 7.06 | 82 | 305 | 0.25 | 0.05 | 2.72 | 0.32 | 8.9 |
| U5 | 0.57 | 20.45 | 134 | 6.15 | 58.7 | 80 | 0.04 | 0.01 | 0.47 | 0.22 | 1.6 |
| U6 | 2.25 | 21.9 | 129 | 6.77 | 40 | 120 | 0.06 | 0.01 | 0.39 | 0.13 | 2.49 |
| A3 | 6.74 | 19.7 | 73 | 7.32 | 170 | 76 | 1.68 | 0.03 | 0.38 | 1.57 | 4.37 |
| A4 | 6.84 | 20.2 | 69 | 7.58 | 190 | 100 | 1.09 | 0.03 | 0.33 | 1.19 | 3.83 |
| A5 | 6.2 | 19.8 | 62 | 7.28 | 297 | 124 | 2.34 | 0.02 | 0.37 | 1.05 | 7.18 |
| A6 | 5.85 | 19.7 | 72 | 7.16 | 290 | 88 | 1 | 0.02 | 0.4 | 1.01 | 5.08 |
| A7 | 6.03 | 21.1 | 100.5 | 7.85 | 94 | 173 | 0.08 | 0.01 | 0.35 | 0.29 | 1.53 |
| A8 | 6.44 | 19.8 | 102 | 7.96 | 211 | 296 | 0.14 | 0.01 | 0.54 | 0.49 | 2.24 |
| A9 | 6.29 | 19.9 | 125 | 7.86 | 88.9 | 79 | 0.33 | 0.01 | 0.51 | 0.02 | 1.27 |
| A10 | 5.89 | 21.8 | 78.4 | 7.24 | 434 | 68 | 0.24 | 0.09 | 0.99 | 1.41 | 8.49 |
| A11 | 5.08 | 20.1 | 143 | 7.57 | 65 | 181 | 0.1 | 0.01 | 0.43 | 0.32 | 1.56 |
| A13 | 7.7 | 18.9 | 62 | 7.39 | 145 | 76 | 0.14 | 0.03 | 0.34 | 1.72 | 9.8 |
| A14 | 7.38 | 20.5 | 62 | 7.45 | 190 | 100 | 0.23 | 0.02 | 0.33 | 1.81 | 5.24 |
| A15 | 6.99 | 22 | 66 | 7.65 | 69.4 | 84 | 0.13 | 0.01 | 0.29 | 1.28 | 3.33 |
| A17 | 7.06 | 27.6 | 367 | 7.98 | 76.5 | 248 | 0.08 | 0.01 | 0.3 | 0.88 | 2.68 |
| A18 | 6.92 | 23 | 67 | 7.77 | 135 | 116 | 0.1 | 0.02 | 0.39 | 1.87 | 5.07 |
| A12 | 7.37 | 18.9 | 71 | 7.39 | 298 | 96 | 0.77 | 0.1 | 0.7 | 1.18 | 6.35 |
| A16 | 7.89 | 18.7 | 66 | 7.79 | 318 | 32 | 0.46 | 0.16 | 0.41 | 1.16 | 4.79 |
